# Hierarchical Porous MoS_2_/C Nanospheres Self-Assembled by Nanosheets with High Electrochemical Energy Storage Performance

**DOI:** 10.1186/s11671-020-03427-5

**Published:** 2020-10-15

**Authors:** Hongdong Liu, Ye Lin, Lei Zhang

**Affiliations:** 1 Engineering Research Center of New Energy Storage Devices and Applications, Chongqing University of Arts and Sciences, Chongqing, 402160 People’s Republic of China; 2 College of Materials Science and Engineering, Chongqing University of Technology, Chongqing, 400054 People’s Republic of China; 3 College of Life Science, Chongqing Normal University, Chongqing, 401331 People’s Republic of China

**Keywords:** Lithium-ion batteries, Anode materials, MoS_2_/C, Porous structure, Electrochemical performance

## Abstract

To overcome the deficiency of the volume expansion of MoS_2_ as the anode material for lithium-ion batteries (LIBs), an effective strategy was developed to design hierarchical porous MoS_2_/carbon nanospheres via a facile, easy-operated hydrothermal method followed by annealing. FESEM and TEM images clearly showed that nanospheres are composed of ultra-thin MoS_2_/C nanosheets coated with carbon layer and possess an expanded interlayer spacing of 0.98 nm. As anodes for LIBs, MoS_2_/carbon nanospheres deliver an initial discharge capacity of 1307.77 mAh g^−1^ at a current density of 0.1 A g^−1^. Moreover, a reversible capacity of 612 mAh g^−1^ was obtained even at 2 A g^−1^ and a capacity retention of 439 mAh g^−1^ after 500 cycles at 1 A g^−1^. The improved electrochemical performance is ascribed to the hierarchical porous structure as well as the intercalation of carbon into lattice spacing of MoS_2_, which offers fast channels for ion/electron transport, relieves the influence of volume change and increases electrical conductivity of electrode. Meanwhile, the expanded interlayer spacing of MoS_2_ in MoS_2_/C can decrease the ion diffusion resistance and alleviate the volumetric expansion during discharge/charge cycles.

## Background

How to protect our sustainable planet while developing and using energy has become a major global issue. In the past decades, lithium-ion batteries (LIBs) highlight the advantages of high energy density, long service life and environmental compatibility, which has great applications in renewable energy storage, electronic devices and energy vehicles [1, 2]. Nevertheless, commercial graphite anodes present a low theoretical specific capacity of 372 mAh g^−1^ [3–5], which is far from the ever-growing requirements for high-energy-density rechargeable batteries. Therefore, it is of great significance to develop desirable electrode materials for LIBs.

Recently, the transition metal sulfides (Co_3_S_4_ [6], SnS_2_ [7], VS_2_ [8], NbS_2_ [9], WS_2_ [10] and MoS_2_ [11]) have been considered a series of potential anode materials owing to their low cost, high energy density and rich redox electrochemistry [12, 13]. Among these materials, molybdenum disulfide (MoS_2_), a semiconductor with a typical two-dimensional (2D) layered structure, has been the most studied material in this family. More importantly, the larger interlayer distance (*d* = 0.62 nm) of MoS_2_ than graphite (*d* = 0.34 nm) can accelerate the process of Li^+^ intercalation/extraction, putting up a high storage capacity of 670 mAh g^−1^ [14–16]. Unfortunately, the practical application of bare MoS_2_ as LIBs is hindered from the poor cycling stability. This is because that the relatively low electronic/ionic conductivities and the electrochemical degradation of the active MoS_2_ materials due to a polysulfide shuttling effect result in the capacity loss and poor rate capability [4, 17, 18]. To tackle these challenges, it has been proven to be particularly effective by engineering various nanostructured MoS_2_ and introducing the conductive carbonaceous materials [15, 19–21]. The essence of the former is to shorten the electronic transmission distance, while the latter aims to improve the overall electronic conductivity of the material, restrain the agglomeration of MoS_2_ and maintain the electrode structure stability, for example, MoS_2_/graphene [22, 23], MoS_2_/CNTs [24], MoS_2_/carbon nanofibers [25], MoS_2_/RGO [26], etc.

Based on the above considerations, recent researches mostly focus on constructing novel MoS_2_/C-based nanocomposites, giving full play to their respective advantages to improve the cycling stability. Li et al. reported a novel 2D MoS_2_/C hybrid nanosheet superstructure consisting of the alternative layer-by-layer interoverlapped single-layer MoS_2_ and mesoporous carbon [4]. The unique hybrid nanosheets with perfect MoS_2_/m-C interface contact result in the maximization of synergistic interaction. Their group also projected 3D ordered macroporous MoS_2_/carbon flexible electrodes by assembling MoS_2_/C nanostructure on carbon cloth with few-layered MoS_2_ nanosheets homogenously embedded into the interconnected carbon wall through the use of polystyrene (PS) nanospheres as the macropore template [17]. The flexible electrodes exhibited superior cycling stability when directly applied for LIBs. Zhang et al. achieved the growth of MoS_2_ nanosheets onto the polypyrrole-derived carbon nanotubes (PCN) substrate and the coating of outer carbon layer onto the nanosheets to fabricate PCN@MoS_2_@carbon sandwiched architecture [27]. In the architecture, ultrathin MoS_2_ nanosheets are sandwiched between hollow PCN and thin carbon layer. Sun et al. successfully prepared 1T-MoS_2_/C hybrids that consist of smaller and fewer-layer MoS_2_ nanosheets via a facile hydrothermal method with a proper glucose additive [28]. The 1T-MoS_2_/C anodes deliver superior cycling stability (maintain 870 mAh g^−1^ after 300 cycles at 1 A g^−1^) and high rate performance (a reversible capacity of 600 mAh g^−1^ at 10 A g^−1^). The superior electrochemical performance can be attributed to the higher intrinsic conductivity of 1T-MoS_2_ and thin carbon layers covered on the surface with an enlarged interlayer spacing of 0.94 nm. Given the above, the modified MoS_2_/C-based nanocomposites can indeed optimizing the electrochemical properties.

Herein, we demonstrate a facile, easy-operated and high effective hydrothermal method toward hierarchical MoS_2_/carbon nanospheres. The nanospheres are self-assembled from ultra-thin MoS_2_/C nanosheets coated with carbon layer, resulting in forming internal interconnected channels and exposing more active sites for electronic/ionic transmission and lithium storage. As a consequence, when employed as anode material in half cell LIBs, the as-prepared open porous structure of MoS_2_/C nanospheres exhibits remarkable lithium storage properties, including high specific capacity, long cycling performance, as well as fast rate capability.

## Methods

### Material Preparation

#### Synthesis of MoS_2_/C

The synthesis of MoS_2_/C nanospheres was based on a previous procedure with modifications [19]. Typically, 0.6 g of sodium molybdate (Na_2_MoO_4_), 3 g of thiourea (CH_4_N_2_S) and 1 g of polyvinyl pyrrolidone (PVP) were dissolved in 30 mL deionized water with magnetic stirring to form a uniform solution. Then, we added 0.2 g of dopamine hydrochloride (DPH) into the above mixture with forming a red suspension. After continuously stirring for 30 min, the resulting suspension was put into a 50-mL Teflon-lined stainless steel autoclave kept at 200 °C for 18 h, followed by cooling down to room temperature naturally. The black precipitates were collected and washed with deionized water and absolute ethanol by centrifugation method, and dried under vacuum at 60 °C for overnight. Lastly, MoS_2_/C nanospheres were obtained by the calcination of black precipitates in argon atmosphere at 700 °C for 3 h. For comparison, we bought commercial pure MoS_2_ powder from Aladdin.

### Material Characterization

The X-ray diffraction (XRD) patterns were measured using a TD-3500 X-ray diffractometer with Cu/Ka radiation (*λ* = 0.15406 nm) at the 2*θ* range of 5°–80° with a scanning rate of 4° min^−1^. N_2_ adsorption/desorption isotherms and Brunauer–Emmett–Teller (BET) surface area were performed by a Micromeritics ASAP 2020 analyzer. The Raman spectrums were tested on a LabRAM HR800 Raman spectrometer equipped with a laser light at 532 nm. The content of carbon in the MoS_2_/C nanospheres was determined by a simultaneous DSC/TGA analyzer (TGA, SDT-Q600) at a heating rate of 10 °C min^−1^ from 25 to 700 °C with a flow of air. The element composition and chemical state of the materials were evaluated through X-ray photoelectron spectroscopy (XPS, Thermo VG ESCALAB 250XI). The detailed morphologies and microstructure of the samples were examined using field emission scanning electron microscopy (FESEM, Sigma 500), and transmission electron microscopy (TEM, Tecnai G2 F20), respectively.

### Electrochemical Tests

The capacities and cycling properties of the as-prepared samples were performed by CR2032 coin-type cells assembled in an argon-filled glove box with lithium sheet as the counter electrode and a Celgard 2400 microporous polypropylene film as the separator. The working electrodes were obtained by mixing in *N*-methyl-2-pyrrolidinone (NMP) solution, the active materials (MoS_2_/C or MoS_2_) with conductive additive (acetylene black) and polymer binder (PVDF) in a 8:1:1 mass ratio to form a homogeneous slurry. We coated the slurry on a copper foil and dried in a vacuum oven at 80 °C for 4 h. Subsequently, the electrodes were punched into circular disks and dried in a vacuum oven at 120 °C for 12 h. 1M LiPF_6_ dissolved in ethylene carbonate (EC) and dimethyl carbonate (DMC) with the volume ratio of 1:1 was selected as the electrolyte. The electrochemical performance was implemented using a battery testing system (Neware BTS-610) in a cut-off voltage window of 0.01–3 V at different current densities. Cyclic voltammetry (CV) curves were carried on an electrochemical workstation (CHI 760E) between 0.01 and 3 V at a scanning rate of 0.2 mV s^−1^.

## Results and Discussion

**Fig. 1:**
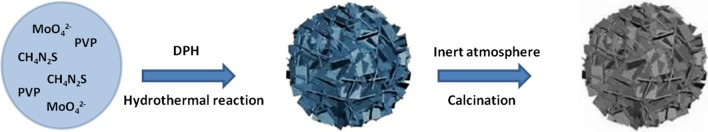
The preparation process diagram of hierarchical porous MoS_2_/C nanospheres

The preparation process diagram of hierarchical porous MoS_2_/C nanospheres is shown in Fig. 1. Na_2_MoO_4_ supplies molybdenum ion, CH_4_N_2_S provides sulfur source, and DPH is carbon source. Polydopamine carbon materials have better electrochemical performance and better conductivity. PVP acts as the dispersant and stabilizer. In hydrothermal process, surfactant PVP preferentially adsorbs along MoS_2_ nanograin boundary, causing MoS_2_ crystal nucleus anisotropic growth and forming MoS_2_ nanosheets. Due to the large specific surface area and high surface energy of MoS_2_ nanosheet/carbon precursor, MoS_2_ nanosheets are self-assembled into nanospheres. Finally, MoS_2_/C nanospheres are prepared by calcination in inert atmosphere.

**Fig. 2:**
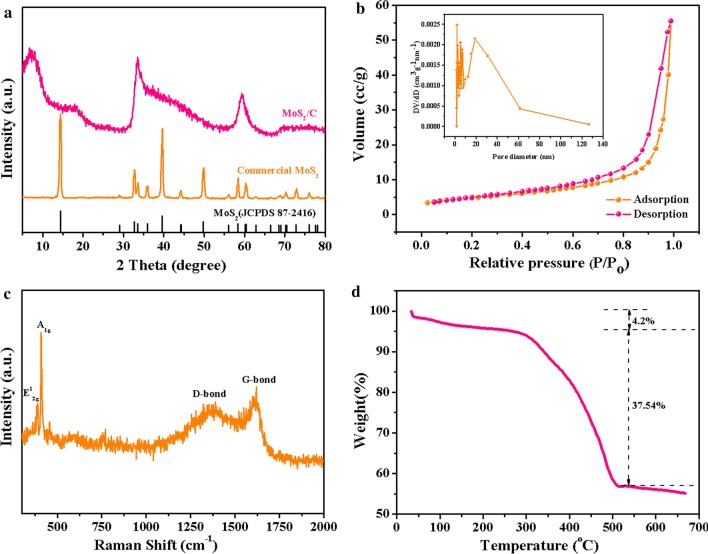
**a** XRD patterns of commercial MoS_2_ and MoS_2_/C nanospheres; **b** N_2_ adsorption/desorption isotherms and pore size distribution of MoS_2_/C nanospheres; **c** the Raman spectrums of MoS_2_/C nanospheres; **d** TGA curve of MoS_2_/C nanospheres

Figure 2a displays the XRD patterns of commercial MoS_2_ and MoS_2_/C nanospheres. The patterns of commercial MoS_2_ are consistent with the standard card of hexagonal 2H-MoS_2_ (JCPDS 87-2416). The diffraction peaks at about 2*θ* = 32.8° and 58.5° can be indexed to (100) and (110) planes of MoS_2_ in both commercial MoS_2_ and as-prepared MoS_2_/C [19, 29]. Additionally, the generation of new structure may be proved by the two new peaks in MoS_2_/C at around 2*θ* = 7.9° and 18.3°, which are pointed to expanded (001) and (002) crystal planes [27]. The expanded d-spacing is also stood by HRTEM observation results (Fig. 2g). The above analysis indicates that we have successfully synthesized MoS_2_/C and the participation of DPH contributed to the expanded interlayer spacing. As shown in Fig. 1b, based on the N_2_ adsorption/desorption isotherms analysis, the BET surface area of MoS_2_/C was concluded to be 16.59 m^2^ g^−1^. The pore size distribution (inset of Fig. 2b) calculated using the BJH equation [2] presents a multi-scale porous structure with an average pore size of 20.66 nm. Such hierarchical porous structure will facilitate the intimate contact between electrode and the electrolyte and fast electron and ion transport, thereby further improving the lithium storage performance. As indicated in Fig. 2c, Raman spectrum of MoS_2_/C confirms the existence and graphitization extent of carbon. Two typical peaks centered at 384 and 407 cm^−1^ are associated with *E*_1_^2g^ and *A*_1g_ vibration modes of MoS_2_ [17, 29, 30], respectively. Another two characteristic peaks located at 1373 cm^−1^ (D band) and 1605 cm^−1^ (G band) can be direct to carbon phase [15, 21, 31]. Detailedly, the D band can be connected with the defective carbon or disordered amorphous carbon due to the *sp*^3^-hybridization on the graphitic plane, while G band is ascribed to crystalline graphitized carbon [19, 32]. The ratio of intensity of D band to G band is calculated to be 0.85, suggesting relatively high graphitization degree of carbon. TGA measurements in Fig. 2d ulteriorly ascertain the carbon content in MoS_2_/C composites. The 4.2% weight loss before 100 °C corresponds to the evaporation of adsorbed free water in the samples, and 37.54% weight loss reveals the oxidation of MoS_2_ to MoO_3_ in air [14, 15]. Hence, the weight percentages of adsorbed water, MoS_2_ and carbon are determined to be 4.2, 64.78 and 31.02 wt%, respectively.

**Fig. 3:**
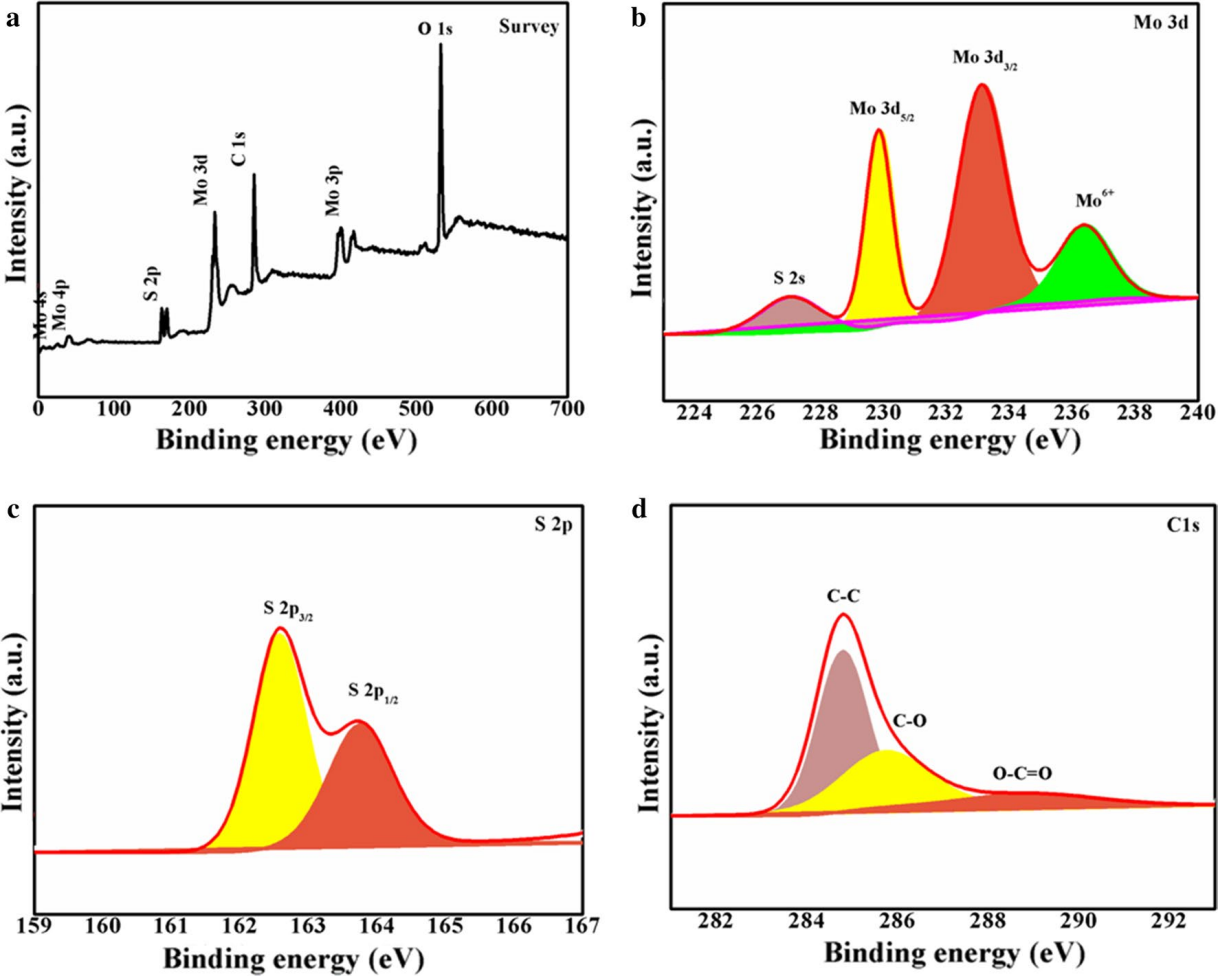
XPS spectrums of MoS_2_/C nanospheres: **a** survey, **b** Mo 3*d*, **c** S 2*p*, **d** C 1*s*

XPS was investigated to assess the element composition and valence states of MoS_2_/C in Fig. 3a–d. As shown in Fig. 3a, the main elements of Mo, S, C and O can be identified from the survey pattern; O derived from the oxygen adsorption on the surface. Figure 3b shows the high-resolution spectrum of Mo 3*d*. Two major peaks centered at 229.8 and 233.1 eV are ascribed to Mo 3*d*_5/2_ and Mo 3*d*_3/2_ of Mo^4+^ in MoS_2_/C [19, 29]. Another broad peaks located at 227.0 eV are generally connected with S 2*s*. And the rest of peak at 236.3 eV can be indexed to Mo^6+^ indicating the formation of C–O–Mo bond between MoS_2_ and carbon, which is consistent with other MoS_2_/C composites [27, 30, 33]. A pair characteristic peaks of S can be clearly observed at 162.5 and 163.7 eV in Fig. 3c, corresponding to S 2*p*_3/2_ and S 2*p*_1/2_ of S^2−^ in MoS_2_[5, 21]. The deconvoluted XPS spectrum of C 1*s* is shown in Fig. 3d; the signals can be fitted to three peaks: The main peaks centered at 284.7 eV correspond to C–C, whereas the two peaks at the 285.6 and 288.9 eV can be assigned to C–O and O–C=O, respectively [14, 29, 34, 35].

**Fig. 4:**
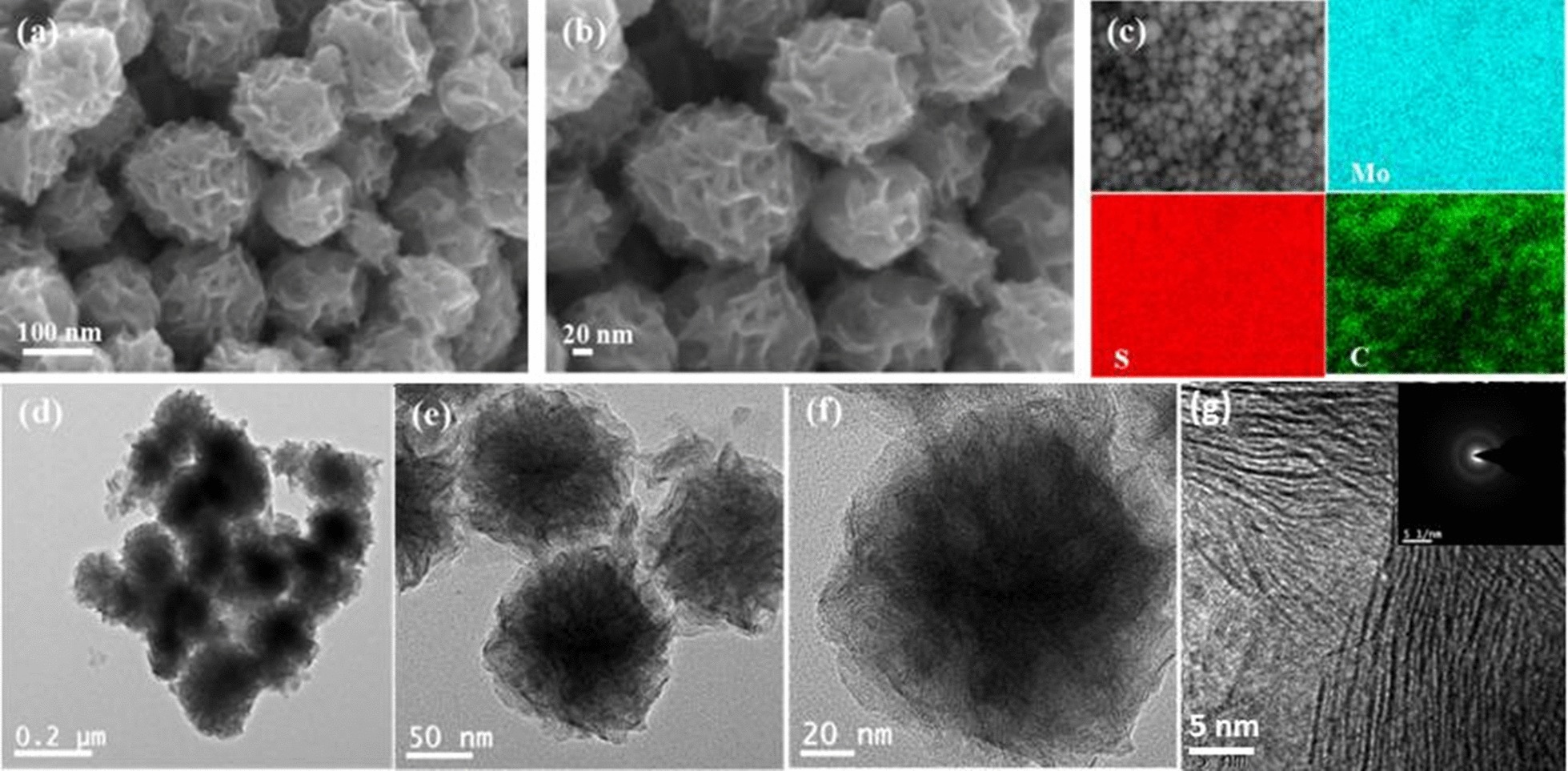
**a**, **b** FESEM images; **c** FESEM district elemental mapping images; **d**–**f** TEM images; **g** HRTEM images and the inset of corresponding SAED pattern of MoS_2_/C nanospheres

As depicted in Fig. 4, FESEM and TEM images disclosed the morphology and structure of MoS_2_/C. The representative FESEM and TEM images of MoS_2_/C are displayed in Fig. 4a–f, revealing porous nanospheres structure assembled from ultra-thin MoS_2_/C nanosheets with an average diameter of 130–200 nm. The FESEM district elemental mapping analysis (Fig. 4c) confirms the existence of Mo, S, C elements and uniform distribution throughout the composites. What is more, the nanosheets are covered with thin carbon layer and closely contact with each other to form a nanosphere in Fig. 4e, f. Such open porous structure is in favor for electronic contact and rapid electron transfer during discharge/charge processes. Figure 4g shows a HRTEM image of MoS_2_/C. The selected area electron diffraction (SAED) pattern indexes a diffraction ring to the polycrystalline nature of the MoS_2_ (inset of Fig. 4g).

**Fig. 5:**
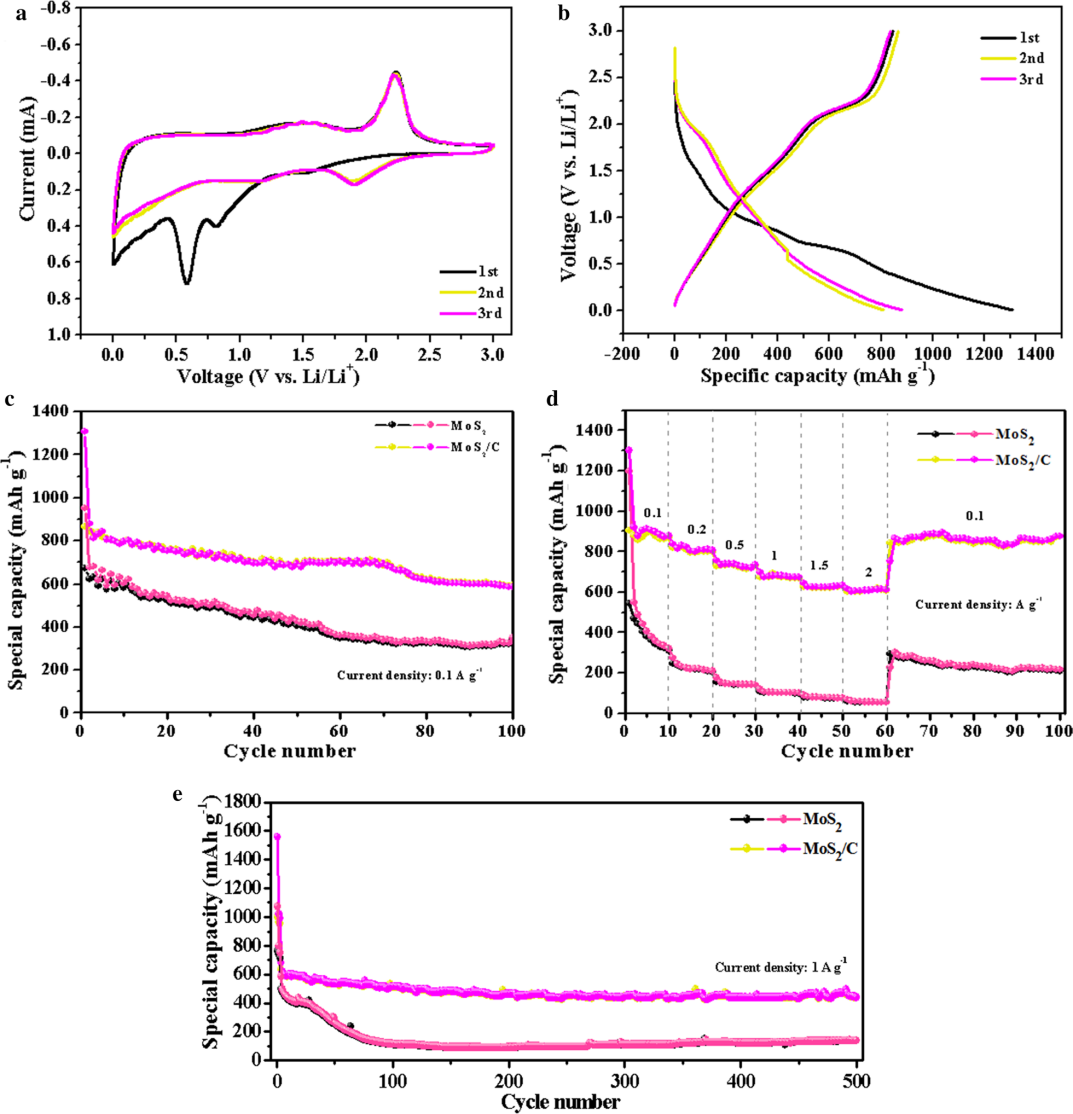
**a** CV curves at scan rate 0.2 mV s^−1^ between 0 and 3 V and **b** discharge/charge profiles at a current density of 0.1 A g^−1^ of MoS_2_/C nanospheres; **c** the cycling performance of MoS_2_ and MoS_2_/C nanospheres at a current density of 0.1 A g^−1^ for 100 cycles; **d** rate performance of two samples at various current rates range from 0.1 to 2 A g^−1^; **e** long-term cycling performance of two samples at a current density of 1 A g^−1^ for 500 cycles

The as-obtained MoS_2_/C composites contain 31.02 wt% of carbon; for comparison, we add the same content of conductive activated carbon powder to the commercial MoS_2_ as the active material, and then mix it with PVDF and acetylene black to gain the electrodes. The two samples were assembled as CR2032 coin-type cells anodes to evaluate their electrochemical mechanism and performance. The first three CV curves of the MoS_2_/C electrode were carried out over the potential window from 0.01 to 3.0 V at a scan rate of 0.2 mV s^−1^. As seen from Fig. 5a, three reduction peaks are observed from 1.22 to 0.1 V in the first cathodic sweep. A broad reduction peak located at 0.73–1.22 V corresponds to the insertion of Li^+^ into the MoS_2_/C and lithiation processes of MoS_2_ to form Li_x_MoS_2_[16, 36]. Another two reduction peaks centered at 0.58 V and 0.1 V can be attributed to the generation of the solid electrolyte interphase (SEI) film and the reduction of Li_*x*_MoS_2_ to Mo [14, 15, 25], respectively. For the first anodic sweep, only two remarkable peaks are noticed at 1.53 and 2.22 V, which are assigned to the oxidation of Mo to MoS_2_ phase and delithiation processes of Li_2_S to S [5, 21, 37]. In the following sweeps, the reduction peak (0.58 V) disappears and other two peaks shifts to 1.17 V and 1.90 V, indicating a multi-lithiation processes of MoS_2_. Notably, the overlapped curves in second/third sweeps mean the high reversibility and great cycling stability of the as-made MoS_2_/C in LIBs. The galvanostatic charge–discharge curves of MoS_2_/C are conducted between 0.01 and 3.0 V at the current density of 0.1 A g^−1^ in Fig. 5b. The arisen charge/discharge voltage platforms are in accordance with the CV results. The MoS_2_/C electrodes deliver discharge capacity as high as 1307.77 mAh g^−1^ and charge capacity of 865.54 mAh g^−1^ with an initial coulombic efficiency (CE) of 66.18%. Moreover, the undesirable CE and capacity loss approximately 33% probably originate from the irreversible decomposition of electrolyte and the generation of SEI film on the electrode surface [5, 14]. The second and third charge/discharge voltage profiles repeat with each other; specific capacities are 845.58/879.20 mAh g^−1^ and 836.13/810.92 mAh g^−1^, respectively. This enhanced CE reveals the electrochemical reversibility of MoS_2_/C anode is good.

Figure 5c compares the cycling performances of the MoS_2_/C and commercial MoS_2_ anodes under the current density of 0.1 A g^−1^ for 100 cycles. Commercial MoS_2_ presents initial charge/discharge specific capacities of 671.70/952.52 mAh g^−1^, which is far from the MoS_2_/C composites (865.54/1307.77 mAh g^−1^). This is because the existence of carbon in MoS_2_/C improve the conductivity and the surface/interfacial storage of Li originated from the porous nanosphere structure and the reversible formation/decomposition of polymeric gel-like film (SEI) [38]. After 100 cycles, the MoS_2_/C and MoS_2_ anodes show discharge specific capacities of 587.18 and 350 mAh g^−1^ with high CE of approximate 99%. The rate capability of two samples was also evaluated at different current densities ranging from 0.1 to 2 A g^−1^ in Fig. 5d. The MoS_2_/C retains high discharge capacities at higher current densities: 878 mAh g^−1^ at 0.1 A g^−1^, 806 mAh g^−1^ at 0.2 A g^−1^, 733 mAh g^−1^ at 0.5 A g^−1^, 673 mAh g^−1^ at 1 A g^−1^, 633 mAh g^−1^ at 1.5 A g^−1^ and 612 mAh g^−1^ at 2 A g^−1^ after 10 cycles. When revaluated at current density of 0.1 A g^−1^, the discharge capacity rapidly reaches 754 mAh g^−1^ and remains 876 mAh g^−1^ after 40 cycles, which is almost close to that of first 10th cycle, suggesting the outstanding rate performance and structural stability of MoS_2_/C. As for MoS_2_, the discharge capacities from 0.1 A g^−1^ to 2 A g^−1^ after 10 cycles are 320 and 55 mAh g^−1^ with enormous capacity loss about 83%. The results reveal that the electrical conductivity of commercial MoS_2_ is no significant improvement due to the addition of activated carbon, thereby not achieving the effect of rapid charge and discharge. This is because simple physical mixing cannot effectively improve the electrical conductivity of commercial MoS_2_, but can accomplish the ideal goal by carbon coating like as-obtained MoS_2_/C.

The long-term cycling performance of two samples is shown in Fig. 5e at a large current density of 1.0 A g^−1^. In order to activate electrodes, the cells are tested at a low current density of 0.05 A g^−1^ for first two cycles. The MoS_2_/C exhibits high discharge capacities of 515, 443 and 439 mAh g^−1^ at 1.0 A g^−1^ for 100th, 300th, 500th cycles, respectively. Compared with the previously reported MoS_2_ anode materials from Table 1, it shows that hierarchical porous MoS_2_/C nanospheres have better electrochemical performance and they will show great potential to replace graphite anode materials. It is worth noting that the capacity curve of MoS_2_/C as a whole is relatively stable without particularly obvious decline except from the first few cycles, illustrating preeminent long-term cycling stability. However, the MoS_2_ anodes suffer from tremendous capacity loss with low discharge capacities of 114, 109 and 138 mAh g^−1^ at the same cycles. As a result, MoS_2_/C still exhibits more superior electrochemical properties than commercial MoS_2_, although activated carbon with the same relative weight ratio is introduced into MoS_2_ electrode. This can be attributed to the following advantages. I. MoS_2_/C composites possess open porous architecture that the nanosphere are self-assembled by ultra-thin MoS_2_/C nanosheets coated with thin carbon layer, which facilitate the intimate contact between electrode and the electrolyte and fast electron and ion transport. Meanwhile, open porous architecture is advantageous to form internal interconnected channels and expose increased number of active sites for electronic/ionic transmission and lithium storage. II. The thin carbon layer derived from carbonization of DPH can not only act as a stable supporting matrix to impede the aggregation of MoS_2_ nanosheets, but also improve the overall conductivity of the material. III. The expanded interlayer spacing of MoS_2_ in MoS_2_/C can decrease the ion diffusion resistance and alleviate the volumetric expansion during discharge/charge cycles.

**Table 1:** Comparison of the electrochemical properties of MoS2

Materials	Current density (mA g^−1^)	Cycle number (*n*)	Reversible capacity (mAh g^−1^)	References
Bulky MoS_2_	200	100	About 100	[39]
MoS_2_	400	30	391	[40]
MoS_2_ nanostructure	100	50	478	[41]
MoS_2_	100	100	479.1	[42]
TiO_2_–MoS_2_ hybrid	800	300	361.5	[43]
MoS_2_/C nanocomposites	100	60	About 450	[44]
MoS_2_/C nanospheres	100	100	587.18	This work
	1000	500	439	

## Conclusions

In this work, we have fabricated hierarchical porous MoS_2_/carbon nanospheres self-assembled by ultra-thin MoS_2_/C nanosheets by a facile hydrothermal method followed by annealing. Benefiting from the rational structure design, the MoS_2_/C provides fast channels for ion/electron transport and maintains high stability and conductivity of the whole electrode in lithium storage. Furthermore, the intercalation of C into interlayer spacing of MoS_2_ can accommodate the volume expansion to make sure the integrality of electrode and enhance electronic conductivity. The as-fabricated MoS_2_/C anode achieves high specific capacity (1307.77 mAh g^−1^ at 0.1 A g^−1^), excellent long cycling performance (439 mAh g^−1^ at 1.0 A g^−1^ for 500 cycles) and fast rate capability (612 mAh g^−1^ at 2 A g^−1^).

## Data Availability

All data are fully available without restriction.

## Abbreviations

LIBs: Lithium-ion batteries
2D: Two-dimensional
PCN: Polypyrrole-derived carbon nanotubes
PS: Polystyrene
DPH: Dopamine hydrochloride
XRD: X-ray diffraction
XPS: X-ray photoelectron spectroscopy
FESEM: Field emission scanning electron microscopy
TEM: Transmission electron microscopy
TGA: Thermogravimetric analysis
BET: Brunauer–Emmett–Teller
BJH: Barrett–Joyner–Halenda
CV: Cyclic voltammetry
DMC: Diethyl carbonate
EC: Ethylene carbonate
PVDF: Polyvinylidene fluoride
